# Immunogenicity Measures of Influenza Vaccines: A Study of 1164 Registered Clinical Trials

**DOI:** 10.3390/vaccines8020325

**Published:** 2020-06-19

**Authors:** Alexander Domnich, Ilaria Manini, Donatella Panatto, Giovanna Elisa Calabrò, Emanuele Montomoli

**Affiliations:** 1 Department of Molecular and Developmental Medicine, University of Siena, Via Aldo Moro 2, 53100 Siena (SI), Italy; ilaria.manini@unisi.it (I.M.); emanuele.montomoli@unisi.it (E.M.); 2 Department of Health Sciences, University of Genoa, Via Pastore 1, 16132 Genova (GE), Italy; panatto@unige.it; 3 Section of Hygiene, Institute of Public Health, Università Cattolica del Sacro Cuore, 00168 Rome, Italy; alisacalabro@icloud.com; 4 VisMederi srl, Strada del Petriccio e Belriguardo 35, 53100 Siena (SI), Italy

**Keywords:** influenza, vaccines, vaccination, influenza vaccines, immunogenicity, correlates of protection, humoral immune response, cellular immune response

## Abstract

Influenza carries an enormous burden each year. Annual influenza vaccination is the best means of reducing this burden. To be clinically effective, influenza vaccines must be immunogenic, and several immunological assays to test their immunogenicity have been developed. This study aimed to describe the patterns of use of the various immunological assays available to measure the influenza vaccine-induced adaptive immune response and to determine its correlates of protection. A total of 76.5% of the studies included in our analysis measured only the humoral immune response. Among these, the hemagglutination-inhibition assay was by far the most widely used. Other, less common, humoral immune response assays were: virus neutralization (21.7%), enzyme-linked immunosorbent (10.1%), single radial hemolysis (4.6%), and assays able to quantify anti-neuraminidase antibodies (1.7%). By contrast, cell-mediated immunity was quantified in only 23.5% of studies. Several variables were significantly associated with the use of single assays. Specifically, some influenza vaccine types (e.g., adjuvanted, live attenuated and cell culture-derived or recombinant), study phase and study sponsorship pattern were usually found to be statistically significant predictors. We discuss the principal findings and make some suggestions from the point of view of the various stakeholders.

## 1. Introduction

Influenza is the leading infectious disease worldwide from the point of view of both attack rate and socioeconomic burden [1]. Together with some general preventive strategies (e.g., frequent hand-washing, social distancing, etc.), influenza vaccination (IV) is a cornerstone public health intervention and can substantially reduce the burden of disease [2].

To be clinically effective, vaccines must, first of all, be immunogenic. Almost all currently available vaccines, including IVs, work through serum or mucosal antibodies that can block infection or bacteremia/viremia; the antibody level therefore predicts protection [3]. In these situations, a precise, widely recognized threshold of the magnitude of antibody levels (as determined through a well-standardized immunological assay) may constitute the so-called “correlate of protection” (CoP). According to a definition by Plotkin [3], a CoP is “*an immune response that is responsible for and statistically interrelated with protection*”.

The immune response to an IV can be measured by means of a variety of immunological assays, the most common being the hemagglutination-inhibition (HAI) assay [4]. The almost ubiquitous use of the HAI assay is probably due to several factors. First of all, this assay is simple, rapid and inexpensive [4,5]. Second, it is/was required by the principal regulatory agencies, such as the United States (US) Food and Drug Administration (FDA) [6] and the European Medicines Agency (EMA) [7], in order to register and/or update annual IV formulations. Third, unlike many other immunological assays, the HAI assay has a universally accepted CoP threshold (≥1:40) that is assumed to indicate a 50% reduction in the risk of acquiring laboratory-confirmed influenza [4,5,6,7]. This estimate comes from an old challenge study conducted in the early ‘70s [8]. More recent studies have tried to verify the previously established ≥1:40 threshold of the HAI titer as a CoP. Some investigations have mostly corroborated this threshold [9,10], while others have underlined the age-dependence of this cut-off [11]. Indeed, it seems that children need a higher HAI titer (at least 1:110) in order to be protected.

Single radial hemolysis (SRH) is another immunological assay used to determine the immunogenicity of IVs. An SRH output of ≥25 mm^2^ lysis zone roughly corresponds to the HAI cut-off titer of ≥1:40 [12]. Of note, the SRH assay has been officially recognized as a CoP by the EMA [7] but not by the FDA [6].

Apart from HAI and SRH, other tests are commonly used and may provide some additional and useful immunogenicity information. Specifically, virus neutralization (VN) assays quantify neutralizing antibodies, are among the only tests that measure the so-called “functional” antibodies directly [5], and may therefore predict vaccine effectiveness better [13]. VN assays, however, are more laborious and time-consuming and less standardized than HAI; moreover, although HAI and VN titers usually show a high correlation, agreement in terms of nominal titers has been found to be relatively low [14]. No universally recognized VN titer for 50% protection has been established so far [15].

The enzyme-linked immunosorbent assay (ELISA), by contrast, is able to measure several class-specific antibodies (including IgM, IgA and IgG) in both serum and oropharyngeal/nasal wash samples [5]. ELISA correlates well with the output of HAI, SRH and VN [5]. However, it has the advantages of: (i) quantifying hemagglutinin (HA) stalk-specific antibodies, and (ii) assessing the immunogenicity of novel universal IV candidates. This is why ELISA is particularly suitable for large studies, as it can yield relatively unbiased results in little time, being fully automatic [5].

The quantification of anti-neuraminidase (NA) antibodies is becoming more common. After HA, NA is the second most abundant surface glycoprotein and allows the virus, in general terms, to spread the viral progeny from the infected host cell to uninfected cells [16]. Nevertheless, the NA antigen has been somewhat neglected in recent decades [17]. However, IVs containing optimal quantities of NA may be particularly useful in order to counteract the phenomena of viral drift and shift, given that NA immunity can offer broader protection [17].

Finally, only the humoral adaptive immunity assays have so far been discussed. However, cell-mediated immunity (CMI) plays an important role in the host immune response in protecting against virus-related illness and in establishing long-term immunological memory. Although correlates of protection are not currently available for CMI, it would be advisable to investigate this kind of immunological response with a view to evaluating next-generation vaccines [18].

The objectives of this study were to summarize current patterns of the use of the various available immunological assays for measuring the IV-induced immune response and to identify the determinants of their use.

## 2. Materials and Methods

### 2.1. Search Strategy, Eligibility Criteria and Data Extraction

In the present study, clinical trials of interest were sought from the freely available US-based prospective registry of studies ClinicalTrials.gov [19], which is the largest in the world.

In order to ensure the maximum number of potentially eligible items, we searched only for the term “Influenza” in the field “Condition or disease”. Adding the more colloquial word “Flu” did not increase the search output. No filters were used.

To be included in the study, clinical trials had both to be composed of vaccinees in at least one study arm and to use at least one immunological assay to quantify IV-induced immunogenicity. Studies were excluded if the actual enrollment was zero, as these trials probably never started. No other restrictions were applied.

The search output (all available columns) was downloaded in a comma-separated values (csv) file on 23 April 2020. Data of interest were then extracted in an ad hoc spreadsheet. If any information was unclear and/or missing, we consulted (whenever possible) publications of the results in peer-reviewed journals that are automatically linked to that particular study by the ClinicalTrials.gov unique identifier.

### 2.2. Study Variables

As per our main objective, the study outcome was the relative distribution of immunological assays used to quantify IV-induced immunogenicity. On the basis of our previous experience, the following outcomes were set a priori: HAI, VN, ELISA, SRH, anti-NA and any CMI assays.

The independent variables of interest were categorized in three domains, namely: spatiotemporal, design-related and IV-related. The first included the start year and location of the study. The study location was categorized in macro-areas as follows: (i) Europe; (ii) US and Canada; (iii) Asia and Pacific; (iv) rest of the world (RoW); and (v) “Multicontinental”. This last category included multicenter trials conducted in different parts of the world.

Attributes regarding the study design included: (i) age-class of the study population; (ii) study type; (iii) study phase; (iv) sponsorship characteristics; and (v) sample size. As per ClinicalTrials.gov, three principle age-groups were defined: children (<18 years), adults (18–64 years) and the elderly (≥65 years). Study type was either interventional or observational, while the study phase was I to IV. Regarding the latter variable, observational research was attributed to phase IV. Studies of phase I/II and II/III were classified as phase II and phase III, respectively. Regarding study sponsorship characteristics, we dichotomized this variable into “industry-sponsored” if a for-profit organization was the study (co)-sponsor, and “non-industry-sponsored” otherwise. The study sample size was the size on enrollment and was readily available in the downloaded file.

The IVs used were classified according to their valence [mono- (usually (pre)-pandemic), tri- and quadrivalent], production platform (traditional egg-based or cell culture-derived/recombinant), presence of adjuvants, mode of administration (e.g., intramuscular/subcutaneous, intradermal, intranasal) and virulence-related issues (inactivated and live attenuated). The inactivated IVs included whole-virion, split or subunit formulations. Moreover, a dummy variable of the universal/supra-seasonal vaccine candidates (e.g., peptide-based, DNA-based) was also created. Virosomal and virus-like particle vaccines were included in the category of adjuvanted IVs [20]. As almost all intranasally administered IVs were live attenuated, these two attributes were analyzed as the single dummy variable “live/intranasal” IVs. Of note, single studies could use different IV types.

### 2.3. Data Analysis

For descriptive purposes, the study outcome of the use of immunological assays was expressed as proportions with 95% confidence intervals (CIs).

In order to identify potential predictors of the use of single immunological assays, we implemented a set of multivariable logistic regression models in which the outcome was a binary variable concerning the use of a given immunological assay (0 = No, 1 = Yes). The potential predictors were the variables described in the above subsection. We implemented both the fully-adjusted models and models selected on the basis of the Bayesian Information Criterion (BIC) minimization approach. The regression outputs were expressed as adjusted odds ratios (aORs) with their corresponding 95% CIs.

In our models, almost all independent variables were dichotomous. The only exception was the study sample size, which was continuous. This latter variable was highly right-skewed (skewness coefficient: 12.7) with an average of 649 (standard deviation: 1855) and a median of 180 (interquartile range: 78–471). For modeling purposes, we split the median. Indeed, the use of the continuous variable worsened the model fits.

*P*-values of < 0.050 were deemed statistically significant. McKelvey–Zavoina’s pseudo-*R*^2^ was used to quantify the explained variability. The Hosmer–Lemeshov test was performed to test the goodness-of-fit. Other model diagnostics included a formal check for multicollinearity; indeed, some potential predictors (e.g., study phase and sample size) could be highly correlated. Multicollinearity was tested by verifying the variance inflation factor (VIF).

All analyses were performed in R stats packages, version 4.0.0 [21].

## 3. Results

### 3.1. Selection of Clinical Trials and Immunological Assays Used

In total, 2294 search items were available on the date of retrieval. Of these, 1186 met the inclusion criteria. Another 22 studies reported zero enrollment and were excluded. Thus, 1164 (50.7%) trials were analyzed.

As expected, the HAI assay was used in the majority (80.6%) of studies, and about half of the studies used only this test. Other, less-commonly used humoral immunity assays were distributed as follows: VN (21.7%), ELISA (10.1%) and SRH (4.6%). Anti-NA antibodies were quantified only in 20 trials (1.7%). CMI was measured in 273 [23.5% (95% CI: 21.1–26.0%)] trials. About 60% of the studies included employed a single assay. Table 1 reports the descriptive statistics on the immunological assays used across the trials.

### 3.2. Determinants of the Immunological Assays Used

As HAI was used in most of the studies included, it was deemed useless to establish its correlates.

The results of the multivariable models predicting the assessment of neutralizing antibodies are reported in Table 2. Compared with the fully-adjusted model, the best-subset model was associated with a significant (−6.5%) BIC reduction; however, the model fit of the latter was poor (Hosmer–Lemeshov test: *p* = 0 043). By contrast, the fully adjusted model fitted well (Hosmer–Lemeshov test: *p* = 0.90); we therefore retained this latter model for interpretation. From the point of view of the vaccine characteristics, monovalent, adjuvanted and cell culture-derived/recombinant IV formulations were significantly associated with a higher use of VN. There was also some increase in VN use as age increased; however, the effect was not significant in the mixed age-classes. Each additional year was associated with about a 7% increase (*p* = 0.016) in the odds of performing a VN. By contrast, studies (co)-sponsored by an industry and those conducted in the RoW were associated with lower odds of using VN assays. Moreover, the later phases of clinical development (phases III and IV) correlated negatively with the use of VN. The model explained 35.3% of variance.

Factors associated with the use of ELISA are described in Table 3. Both models showed similar results, fit reasonably well (Hosmer–Lemeshov test: *p* ≥ 0.80) and explained up to 42% of variance. Live attenuated/intransal (OR = 8.28) and cell-derived/recombinant (OR = 2.41) IVs were positively associated with ELISA testing. By contrast, the use of adjuvanted IV formulations was a significant negative predictor. Compared with phase I clinical trials, those of phases II to IV were associated with a 64–90% lower rate of ELISA testing. Analogously, trials involving industry made less use of ELISA.

CMI was among the outcomes in 273 of the trials included [23.5% (95% CI: 21.1–26.0%)]. The results of the adjusted logistic models with the outcome of CMI assays are reported in Table 4. On the basis of goodness-of-fit, only the fully adjusted model was retained (Hosmer–Lemeshov test: *p* = 0.61) since the best-subset model proved to have a poor fit (Hosmer–Lemeshov test: *p* = 0.002). Trials investigating adjuvanted, live attenuated/intranasal IVs and, especially, universal vaccine candidates displayed significantly higher odds of quantifying CMI. As in the previously described models, industry co-sponsored trials and phase III studies reported a lower use of CMI assays. Moreover, larger studies were also associated with 38% lower odds of the model outcome.

In none of the models reported in Table 2, Table 3 and Table 4 did multicollinearity issues emerge: no VIF exceeded the nominal value of 5.

Finally, the outcomes of SRH and anti-NA assay use were relatively infrequent, and thus displayed a low event-per-predictor ratio. We therefore decided against the multivariable regression approach. However, two notable features regarding the SRH assay emerged: (i) all [100% (95% CI: 93.4–100%)] studies were industry sponsored and (ii) two thirds [66.7% (95% CI: 52.5–78.9%)] were conducted in Europe. By contrast, most [65.0% (95% CI: 40.8–84.6%)] anti-NA antibody testing was performed in the US.

## 4. Discussion

To our knowledge, the present paper is the first to describe and analyze the use of the available immunological assays for quantifying the immunogenicity of the currently licensed IVs and vaccine candidates. Several findings emerged from the present analysis. First (and not to our surprise), we found that most of the studies included used only one assay, which in most cases was the HAI. Second, we found that some IV formulations and some study design attributes, such as phase or sponsorship, were associated with the patterns of use of particular immunological assays. We will now discuss our principal findings.

Regarding IV valence characteristics, we did not generally find any meaningful correlation, the only exception being the significantly higher probability of neutralizing antibody quantification in trials involving monovalent IVs. In our study, most monovalent IVs were either pandemic A(H1N1)pdm09 or pre-pandemic vaccines against several avian type A subtypes with pandemic potential [e.g., A(H5N1), A(H7N9)]. Indeed, VN has proved to be particularly useful in studying the serology of avian type A strains, and several studies have documented the unsuitability of HAI for the detection of antibodies against these viruses [22,23,24]. Moreover, the pattern observed may be somehow linked to antigen-sparing as a strategy for pandemic preparedness promoted by the World Health Organization [25]. Indeed, in the present analysis, many (pre)-/pandemic studies were dose-finding. Compared with HAI, VN can detect antibodies at lower titers, distinguish better between small differences (e.g., less than two-fold) in pre- and post-vaccination titers, and requires a lower concentration in order to yield a judgment of protection (though no formally established threshold has been universally recognized) [24,26,27,28,29].

Adjuvanted IVs (including virosomal and virus-like particle formulations) were associated with a higher use of both VN and CMI assays. Adjuvanted IVs have been systematically shown to induce both stronger and broader humoral immune responses [30,31]. However, widespread use of the standard HAI assay may downplay some important potential advantages of the adjuvanted formulations, such as cross-protection and immunological memory. According to the FDA’s Center for Biologics Evaluation and Research (CBER) guidelines [6] despite the public health advantage of adjuvants in terms of dose-sparing, some safety issues may arise; however, the subsequent risk–benefit assessment asserts that “*meaningful differences may also include a demonstration of cross-reactivity against drifted strains*” [6]. In such situations, VN assays may provide an advantage: they identify a wide range of antibodies, including those that neutralize the virus by inhibiting its entry/replication in mammalian cells, while HAI only measures antibodies against HA, which act by preventing the agglutination of red blood cells [24]. Ansaldi et al. [32], for example, showed that in elderly subjects immunized with an adjuvanted trivalent IV, the correlation coefficient *r* between the mean-fold increase in neutralizing antibody titers (from pre- to post-vaccination) and the antigenic distance of several drifted A(H3N2) strains was substantially higher than the *r* between the corresponding mean-fold increase in HAI titers (0.701 vs. 0.501). Analogously, the use of CMI, which plays a crucial role in protecting against influenza by establishing the long-term immunological memory [18], may also positively “differentiate” the adjuvanted formulations from their non-adjuvanted counterparts. For instance, Zedda et al. [33] found that adding an adjuvant to standard IVs induced a larger expansion of vaccine-specific CD4+ cells, and that this advantage was evident with regard to the drifted heterologous strains. To summarize, our results suggest that measuring neutralizing antibodies and CMI may constitute the so-called “correlates of adjuvanticity” [34]; it is therefore advisable to better standardize protocols for these assays, in order to reduce intra- and inter-laboratory variation, and to revise the current immunogenicity guidelines [24].

Intranasal IV formulations (mostly live attenuated) proved more likely to be tested in ELISA, with a huge effect size of 8.3. The use of intranasal IV formulations also was also seen to be a positive predictor of CMI assays. The standard HAI assay is often judged poorly suitable for live attenuated IVs [24] and, unlike in the case of inactivated IVs, no CoPs have been established for live IVs [35]. For instance, in their challenge study, Wright et al. [36] showed that some traditional measurements of immune response, such as HAI, did not correlate with protection provided by a live attenuated IV. Indeed, live attenuated IV formulations are believed to induce multifaceted immunogenicity ascribable to both local/mucosal immunoglobulins and T cell responses [37]. In an analysis of three clinical studies, Ambrose et al. [38] found that nasal wash IgA measured by means of ELISA contributed to the efficacy of live IV in young children. Nasal wash IgG and IgM may also increase in recipients of live IV [38]. With regard to CMI, Forrest et al. [39] showed that this may have greater importance in subjects immunized with live IVs in comparison with inactivated formulations. In their study, it was also estimated that the majority of children with ≥ 100 interferon-*γ* spot-forming cells per 10^6^ peripheral blood mononuclear cells were protected against clinical influenza, suggesting that this level could be a possible target in clinical trials. As discussed by Ambrose et al. [38], in Plotkin’s framework of CoPs [3], the association between the protection induced by live IV and IgA responses measured by means of ELISA constitutes the so-called co-correlate of protection, which is “*one of two or more factors that correlate with protection in alternative, additive, or synergistic ways*” [3]. Indeed, strain-specific IgA responses are associated with protection in vaccinees, but the level of response may vary by strain and trial, and IV-induced protection may be correlated with other components of the immune response [38].

Universal vaccine candidates have likewise displayed far greater odds of being scrutinized through CMI assays. Universal IVs comprise a large and heterogeneous variety of experimental vaccine formulations with different platforms, targets and mechanisms of action [40,41,42]. As we have already mentioned, the currently accepted CoP assays of HAI and SRH target the viral HA. By contrast, most next-generation universal IVs target some highly conserved proteins that are common across viral (sub) types [40,41,42]. This is why the recognized CoPs and other widely used immunological assays are likely to prove unsuitable for the universal vaccine candidates. On the other hand, CMI will undoubtedly be quantified in future trials on the next-generation IVs, since cross-reactive CD4+ and CD8+ T cells have already been proposed as future CoPs in human challenge and cohort studies [43].

Unlike the other tests analyzed, and independently from the IV formulations used, VN assays were employed increasingly over the 20-year period considered. As described earlier, the proliferating interest in quantifying neutralizing antibodies is probably determined by the fact that, unlike the conventional assays, VN tests measure the functional capability of antibodies and not just their total quantity, and are more efficient in quantifying cross-reactivity/cross-immunogenicity.

Industry-(co)sponsored trials quantified neutralizing and ELISA antibodies and determined CMI to a significantly lesser extent than non-industry-sponsored studies. By contrast, about 85% of industry-(co)sponsored studies determined the HAI response, while 100% of studies that used the SRH assay were industry-(co)sponsored. The most probable explanation is two-fold: (i) both HAI and SRH have a well-recognized threshold as a CoP [9,10,11,12] and (ii) clinical guidelines are available for industry to support the licensure of seasonal IVs [6,7]. Indeed, the US document issued by the CBER [6] cites some criteria to support accelerated approval of new IVs, and all these criteria are based on the HAI assay. Indeed, in the case of children and adults aged <65 years, clinical trials should show that the lower limit of the two-sided 95% CI for the percentage of subjects achieving seroconversion (defined as the proportion of vaccinees with at least a four-fold increase from before to after IV) and seroprotection (defined as the proportion of vaccinees with an HAI titer ≥ 1:40) reaches or exceeds 40% and 70%, respectively. In the case of elderly subjects, these proportions are reduced by 10% (to 30% and 60%, respectively) [6]. The previous European criteria issued by the EMA’s Committee for Medicinal Products for Human Use [7] were similar, but were based on the point estimates rather than the 95% CIs. Moreover, unlike in the US guidelines, the endpoints determined by the SRH assay were recommended only from the European perspective [7]; this is probably why we found that most SRH assays were performed in Europe.

In all our analyses, a later phase of clinical development was generally associated with a lower use of VN, ELISA and CMI assays. Contrary to our expectations, we did not encounter any problem of collinearity between the study phase and sample size; an adequately powered sample size is usually directly related to the study phase. The observed absence of collinearity issues was probably driven by the sample size dichotomization rule adopted. Early phase trials on vaccines usually have safety endpoints as primary outcomes; however, some exploratory immunogenicity endpoints may also be assessed (usually as secondary outcomes). By contrast, pivotal phase III trials are designed to provide robust clinical data in support of licensure [44,45]. Modern phase III immunogenicity trials enroll thousands of individuals, each of whom is tested for the IV-induced immune response at least twice. In these conditions, the HAI assay was most frequently used, not least because this test is both widely recognized as a CoP and relatively cheap [5]. Indeed, the reproducibility and unbiased assessment of an assay should be weighed against its cost-effectiveness [14]. On the other hand, the EMA guidelines [7] state that “*It is essential that neutralizing antibody titers are determined in all studies*”, “*Measurement of … CMI is encouraged*” and “*Applicants may consider evaluating anti-NA antibodies*”. We therefore believe that more sophisticated techniques should also be used in at least a subset of participants in pivotal clinical trials.

Despite its strengths—such as its large sample size and meaningful set of predictors—the current study may have some notable limitations. First of all, the information retrieved came mostly from the registered information on clinical trials and is therefore is highly dependent on the quality of this latter. In this regard, Viergever et al. [46] have shown that the quality of registration at ClinicalTrials.gov is suboptimal, although some slight improvements have been seen over time. We tried to attenuate this bias by consulting the available peer-reviewed publications linked to a given trial; however, this was not always possible. Indeed, in 11.4% of the trials included, we failed to identify the humoral immunity assays used (although we believe that most of these used the HAI assay). This is also why we cannot completely rule out mistakes due to misclassification bias of the study outcome. For instance, records indicating that the immune response was measured according to the CBER criteria were assumed to refer to studies that used the HAI assay, given that the US criteria consider only the HAI assay [6].

Second, although ClinicalTrials.gov is the world’s largest and first-established registry, we acknowledge that a certain number of studies were registered in other supranational (e.g., the European register available at www.clinicaltrialsregister.eu) or country-specific databases, which were not searched systematically. At present, it is not possible to perform a simultaneous search in more databases several databases nor can double-registered trials be directly linked via a single identifier (in order to avoid duplicates). We believe, however, that the sample of registered trials analyzed is globally representative, given the pioneering nature of ClinicalTrials.gov [19]. We also believe that this shortcoming is particularly relevant with regard to the non-industry-sponsored trials; indeed, in our sample approximately 62% of items were industry-(co)sponsored. Vaccine manufacturers are obliged to prospectively register their trials, and the technical documents commonly submitted to the regulatory agencies have to contain a complete list of the studies that support marketing authorization applications [47].

Third, considering the relative paucity of studies that measured CMI, no attempt to further categorize CMI assays was made. It is therefore probable that some meaningful associations were “hidden” by the classification rule adopted.

Finally, we were not able to identify determinants of the SRH and anti-NA assays, owing to the paucity of studies using these tests. Indeed, according to a widely applied “rule of thumb” [48], we needed at least 10 events per independent variable. We tried to address this issue by applying Firth’s penalized logistic regression approach; the output was, however, not consistent (results not shown) [49].

## 5. Conclusions

In conclusion, the IV-induced immune response may be measured by means of a variety of immunological assays; these are, however, unevenly distributed across the available registered trials. Continuous diversification of the IV market and research into a universal IV will probably produce a gradual shift from the currently preferred HAI test to other more “functional” assays; assays that measure CMI seem particularly promising. Technological innovation can involve high costs and exerts strong financial pressure on health systems. Today’s healthcare systems cannot forgo technological innovation, but must take into account the point of view of the various stakeholders: patients should be guaranteed rapid access to more effective healthcare technologies; research and development efforts should be encouraged when oriented towards the production of high-value products; institutions and regulatory agencies should support innovation by using evidence-based tools for their evaluation, such as health technology assessment (HTA); and health systems should promote technological innovation while ensuring their own sustainability [50]. In this regard, governments around the world are increasingly focusing on the use of public-private partnerships that can combine the strengths of private enterprise, such as innovation, technical knowledge and managerial skills, with the role of public institutions, including social responsibility and public accountability, in order to deliver high-quality health services [51]. Future IV clinical trials will undoubtedly benefit from functional public–private partnerships, especially from the point of view of searching for new CoPs.

## Figures and Tables

**Table 1:** Frequency of the immunological assays used in the clinical trials included (*N* = 1164).

Parameter	% (*N*)	95% CI
Humoral response only	76.5 (891)	74.0–79.0
Cell-mediated response only	3.0 (35)	2.1–4.2
One assay only	61.3 (714)	58.5–64.2
Two assays	26.8 (312)	24.3–29.4
Three assays or more	11.9 (138)	10.1–13.9
HAI assay	80.6 (938)	78.2–82.8
HAI assay only	47.8 (556)	44.9–50.7
VN assays	21.7 (253)	19.4–24.2
VN assays only	1.0 (12)	0.5–1.8
ELISA	10.1 (117)	8.4–11.9
ELISA only	0.9 (10)	0.4–1.6
SRH assay	4.6 (54)	3.5–6.0
SRH assay only	1.2 (14)	0.7–2.0
Anti-NA response	1.7 (20)	1.1–2.6
Anti-NA response only	0 (0)	0.0–0.3
Humoral response assay unclear	11.4 (133)	9.7–13.4

ELISA: enzyme-linked immunosorbent assay; HAI: hemagglutination-inhibition; NA: neuraminidase; SRH: single radial hemolysis; VN: virus neutralization.

**Table 2:** Multivariable logistic regression models to predict the use of immunological assays measuring neutralizing antibodies.

Variable	Level	Best-Subset Model		Full Model	
		aOR (95% CI)	*P*	aOR (95% CI)	*P*
Vaccine	Monovalent ^a^	5.68 (3.89–8.30)	<0.001 ***	3.35 (1.75–6.44)	<0.001 ***
	Trivalent ^a^	–	–	0.70 (0.41–1.22)	0.21
	Quadrivalent	–	–	1.07 (0.57–1.98)	0.84
	Adjuvanted ^a^	1.48 (1.02–2.15)	0.038 *	1.65 (1.10–2.46)	0.015 *
	Intradermal ^a^	–	–	1.04 (0.45–2.37)	0.93
	Live/intranasal ^a^	–	–	1.43 (0.81–2.54)	0.22
	Cell-derived/recombinant ^a^	2.03 (1.37–3.00)	<0.001 ***	1.82 (1.18–2.80)	0.006 **
	Universal candidates ^a^	–	–	0.55 (0.19–1.63)	0.28
Age	Any	Ref	–	Ref	–
	Children only	3.49 (1.22–9.99)	0.020 *	2.92 (0.97–8.83)	0.058
	Adults only	3.59 (1.32–9.75)	0.012 *	2.90 (1.01–8.34)	0.048 *
	Elderly only	5.58 (1.85–16.89)	0.002 **	4.71 (1.49–14.88)	0.008 **
	Children and adults	1.83 (0.52–6.42)	0.35	1.68 (0.45–6.22)	0.44
	Adults and elderly	1.67 (0.60–4.65)	0.33	1.59 (0.54–4.66)	0..40
Study type	Observational	–	–	Ref	–
	Interventional	–	–	0.63 (0.26–1.49)	0.29
Study phase	1	–	–	Ref	–
	2	–	–	0.72 (0.45–1.16)	0.17
	3	–	–	0.49 (0.26–0.90)	0.022 *
	4	–	–	0.36 (0.20–0.64)	<0.001 ***
Industry sponsored	No	Ref	–	Ref	–
	Yes	0.48 (0.33–0.68)	<0.001 ***	0.40 (0.26–0.90)	<0.001 ***
Time	Year ^b^	1.08 (1.04–1.13)	<0.001 ***	1.07 (1.01–1.12)	0.016 *
Sample size	<180	–	–	Ref	–
	≥180	–	–	1.27 (0.87–1.86)	0.21
Study location	Multicontinental	–	–	Ref	–
	Europe	–	–	0.67 (0.28–1.58)	0.36
	US and Canada	–	–	0.62 (0.27–1.46)	0.27
	Asia and Pacific	–	–	0.85 (0.36–2.04)	0.72
	Rest of the world	–	–	0.09 (0.02–0.49)	0.006 **
Pseudo-*R*^2^, %		30.0		35.3	
BIC		1008.6		1074.2	

^a^ Yes versus No; ^b^ One-year increase; *** *p* < 0.001; ** *p* < 0.01; * *p* < 0.05; *p* < 0.10; aOR: adjusted odds ratio; BIC: Bayesian information criterion.

**Table 3:** Multivariable logistic regression models to predict the use of enzyme-linked immunosorbent assay (ELISA).

Variable	Level	Best-Subset Model		Full Model	
		aOR (95% CI)	*P*	aOR (95% CI)	*P*
Vaccine	Monovalent ^a^	–	–	1.04 (0.37–2.96)	0.94
	Trivalent ^a^	–	–	1.62 (0.65–4.04)	0.30
	Quadrivalent	–	–	0.82 (0.34–2.01)	0.67
	Adjuvanted ^a^	0.34 (0.17–0.67)	0.002 **	0.32 (0.15–0.65)	0.002 **
	Intradermal ^a^	–	–	0.30 (0.06–1.37)	0.12
	Live/intranasal ^a^	8.28 (4.86–14.11)	<0.001 ***	8.60 (4.75–15.56)	<0.001 ***
	Cell-derived/recombinant ^a^	2.41 (1.37–4.24)	0.002 **	2.28 (1.19–4.37)	0.013 *
	Universal candidates ^a^	–	–	1.97 (0.57–6.80)	0.28
Age	Any	–	–	Ref	–
	Children only	–	–	1.40 (0.33–6.02)	0.65
	Adults only	–	–	1.34 (0.34–5.35)	0.68
	Elderly only	–	–	2.62 (0.56–12.29)	0.22
	Children and adults	–	–	1.26 (0.25–6.33)	0.78
	Adults and elderly	–	–	1.06 (0.26–4.35)	0.94
Study type	Observational	–	–	Ref	–
	Interventional	–	–	0.57 (0.21–1.55)	0.27
Study phase	1	Ref	–	Ref	–
	2	0.36 (0.19–0.66)	0.001 **	0.37 (0.19–0.71)	0.003 **
	3	0.10 (0.04–0.29)	<0.001 ***	0.12 (0.04–0.36)	<0..001 ***
	4	0.32 (0.18–0.59)	<0.001 ***	0.28 (0.14–0.59)	<0.001 ***
Industry sponsored	No	Ref	–	Ref	–
	Yes	0.45 (0.27–0.75)	0.002 **	0.46 (0.27–0.81)	0.007 **
Time	Year ^b^	–	–	1.06 (0.99–1.14)	0.090
Sample size	<180	–	–	Ref	–
	≥180	–	–	0.72 (0.41–1.27)	0.26
Study location	Multicontinental	–	–	Ref	–
	Europe	–	–	1.89 (0.22–16.63)	0.57
	US and Canada	–	–	1.16 (0.13–10.10)	0.90
	Asia and Pacific	–	–	1.02 (0.11–9.36)	0.98
	Rest of the world	–	–	0.89 (0.06–12.55)	0.93
Pseudo-*R*^2^, %		38.3		42.4	
BIC		571.3		666.9	

^a^ Yes versus No; ^b^ One-year increase; *** *p* < 0.001; ** *p* < 0.01; * *p* < 0.05; *p* < 0.10; aOR: adjusted odds ratio; BIC: Bayesian information criterion.

**Table 4:** Multivariable logistic regression models to predict the use of immunological assays measuring cell-mediated immunity.

Variable	Level	Best-Subset Model		Full Model	
		aOR (95% CI)	*P*	aOR (95% CI)	*P*
Vaccine	Monovalent ^a^	–	–	1.48 (0.79–2.78)	0.22
	Trivalent ^a^	–	–	1.56 (0.90–2.70)	0.11
	Quadrivalent	–	–	1.76 (0.99–3.14)	0.055
	Adjuvanted ^a^	1.56 (1.09–2.23)	0.014 *	1.53 (1.03–2.29)	0.035 *
	Intradermal ^a^	–	–	1.15 (0.58–2.27)	0.69
	Live/intranasal ^a^	2.56 (1.65–3.96)	<0.001 ***	2.66 (1.65–4.29)	<0.001 ***
	Cell-derived/recombinant ^a^	–	–	1.35 (0.86–2.12)	0.20
	Universal candidates ^a^	10.10 (4.17–24.19)	<.001 ***	10.05 (4.17–24.19)	<0.001 ***
Age	Any	–	–	Ref	–
	Children only	–	–	0.63 (0.27–1.48)	0.29
	Adults only	–	–	1.16 (0.55–2.47)	0.70
	Elderly only	–	–	0.92 (0.38–2.26)	0.86
	Children and adults	–	–	0.60 (0.22–1.63)	0.32
	Adults and elderly	–	–	0.83 (0.40–1.76)	0.63
Study type	Observational	–	–	Ref	–
	Interventional	–	–	0.64 (0.35–1.17)	0.15
Study phase	1	Ref	–	Ref	–
	2	0.56 (0.37–0.87)	0.009 **	0.72 (0.45–1.14)	0.16
	3	0.32 (0.18–0.56)	<0.001 ***	0.47 (0.25–0.90)	0.022 *
	4	0.94 (0.62–1.42)	0.76	1.17 (0.70–1.95)	0.54
Industry sponsored	No	Ref	–	Ref	–
	Yes	0.32 (0.23–0.45)	<0.001 ***	0.31 (0.21–0.46)	<0.001 ***
Time	Year ^b^	–	–	0.99 (0.94–1.03)	0.60
Sample size	<180	–	–	Ref	–
	≥180	–	–	0.62 (0.44–0.88)	0.007 **
Study location	Multicontinental	–	–	Ref	–
	Europe	–	–	3.95 (0.84–18.54)	0.08
	US and Canada	–	–	2.28 (0.49–10.68)	0.30
	Asia and Pacific	–	–	1.68 (0.35–8.14)	0.52
	Rest of the world	–	–	2.32 (0.41–13.31)	0.34
Pseudo-*R*^2^, %		23.4		29.5	
BIC		1144.4		1224.4	

^a^ Yes versus No; ^b^ One-year increase; *** *p* < 0.001; ** *p* < 0.01; * *p* < 0.05; *p* < 0.10; aOR: adjusted odds ratio; BIC: Bayesian information criterion.
